# Detection of partial and/or complete Y chromosome microdeletions of azoospermia factor a (AZFa) sub‐region in infertile Iraqi patients with azoospermia and severe oligozoospermia

**DOI:** 10.1002/jcla.24272

**Published:** 2022-02-05

**Authors:** Mushtak T. S. Al‐Ouqaili, Sahar K. Al‐Ani, Rehab Alaany, Mohammed N. Al‐Qaisi

**Affiliations:** ^1^ Department of Microbiology College of Medicine University of Anbar Al‐Anbar Governorate Ramadi Iraq; ^2^ Ministry of Health Al‐Anbar Health Office Al‐Anbar Governorate Ramadi Iraq

**Keywords:** complete AZF microdeletion, male infertility, partial AZFa/ microdeletion, Y/chromosome/microdeletion

## Abstract

**Background:**

This study aimed to analyze the incidence of azoospermia factor a (AZFa) microdeletions in the Y chromosome and their association with male infertility in a population with azoospermia and severe oligozoospermia from Iraq.

**Methods:**

A total of 75 infertile Iraqi males and 25 healthy controls were included in this study. The semen analysis was performed to determine the azoospermia, severe oligozoospermia, or normal cases. The AZFa microdeletions were investigated using the real‐time polymerase chain reaction (real‐time PCR). Then, AZFa sub‐region deletions were investigated by a conventional PCR.

**Results:**

In total, 40 men with azoospermia and 35 men with severe oligozoospermia were selected. Out of 75 infertile males, 46 (61.3%) individuals had AZFa microdeletions, of whom 32 (69.6%) had partial deletion, while 14 (30.4%) males had complete deletion using real‐time PCR. The frequency of microdeletions was significantly different between the infertile and control group (*p*‐value < 0.00001). The proportion of AZFa microdeletions appeared higher in azoospermia men (72.5%, *n* = 29/40) than severe oligozoospermia men (48.6%, *n* = 17/35), but based on the conventional PCR results, only one azoospermia patient (2.2%) was shown to have complete AZFa deletion, while the other 45 patients (97.8%) had partial AZFa deletions.

**Conclusion:**

In this study, the partial AZFa microdeletions were more numerous than complete AZFa deletion. According to our results, the AZFa microdeletions might be associated with male infertility and spermatogenic failure. It is recommended to investigate the AZFa sub‐region microdeletions in patients that shown AZFa microdeletions in primary screening.

## Graphical abstract

In total, 75 infertile Iraqi were investigated by real‐time PCR and PCR for azoospermia factor a (AZFa) microdeletions. Out of 75 infertile males, 46 (61.3%) individuals had AZFa microdeletions, of whom one patient (2.2%) was shown to have complete AZFa deletion, while the other 45 patients (97.8%) had partial AZFa deletions.

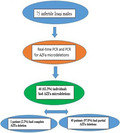

## 1 INTRODUCTION

Tiepolo and Zuffardi^1^ were the first to identify correlations between Y chromosome deletions and male infertility. The Y chromosome is a sex chromosome and is one of the shortest human genome chromosomes (~50 million bp), representing about 2%–3% of the haploid genome. The human Y chromosome is recognized as a pauper gene, but it plays an important rᴏle.in human reproduction as its presence or absence determines gonadal sex. The Y chromosome often plays a major role in the control of sperm formation and male infertility. It occupies a prime place in the human genome because of its organization, and job. The cytogenetic design of the Y chromosome is acrocentric, and it has a small arm (known as Yp), and a long arm (known as Yq), clearly differentiated by the centromere‐region, that is necessary for chromosomal separation in male meiosis.

The human Y chromosome is essential for human sex determination and male germ cell development and maintenance.^2^ The azoospermia factor (AZF) region is located ᴏn the long arm ᴏf the Y chromosome Yq. It plays a vital role in the genetics ᴏf male infertility and is divided into three sub‐regions: AZFa, AZFb, and AZFc. These regions contain genes that are involved in spermatogenesis and the development ᴏf testes. Microdeletions in this region lead to spermatogenetic defects and male infertility.^3^ The AZFa sub‐region is located in the proximal region in the long arm ᴏf the Y chromosome Yq. Four various genes have been recognized in this region, including ubiquitin‐specific peptidase 9 Y (USP9Y) chromosome gene, previously known as DFFRY or Drosophila fat facets related Y gene; DEAD box RNA helicases, Box 3, Y‐linked (DDX3Y or DBY); ubiquitously transcribed tetratricopeptide repeat containing, Y‐linked (UTY); and thymosin beta 4 Y‐linked (TB4Y) gene. The main gene of the AZFa region is DDX3Y, which is expressed in the testis and is involved in the progression of pre‐meiotic germ cells, indicating that inhibiting DDX3Y expression plays a role in prenatal germ cell depletion and hence infertility. The USP9Y gene also assists in spermatogenesis.^4^, ^5^ Azoospermia, oligozoospermia, and oligoasthenozoospermia are caused by shortening or loss of the USP9Y gene.^6^ Generally, removals of the AZFa region that delete both of these genes result in sertoli cell‐only syndrome (SCOS), a state characterized not only by the presence of full sertoli cells in the testes but also by the absence of spermatozoa and azoospermia.^7^ However, limited AZFa removals are correlated with phenotypes ranging from azoospermia to normozoospermia.^8^ Thus, a diagnosis of total deletion of the AZFa area means that it is virtually impossible for testicular sperm through intra‐cytoplasmic sperm injections (ICSI).

In a recent study by Al‐Janabi et al.^9^ from Iraq, the most predominant deleted region was AZFb (33.3%), followed by AZFc region (23.0%), while no microdeletion was noticed in AZFa. In severe oligozoospermia and azoospermia men, the incidence of the microdeletions in AZFa, AZFb, and AZFc regions ranged from 1% to 50.0%. In around 15.0% of couples who want to have a child, infertility is a public health concern; the male gender is present in approximately 50.0% of instances.^9^ So far, few studies have been conducted on the prevalence of AZFa microdeletions in azoospermia and severe oligozoospermia infertile Iraqi patients. Hence, this study aimed to analyze the incidence of AZFa microdeletions in the Y chromosome in patients with azoospermia and severe oligozoospermia from Iraq.

## 2 MATERIALS AND METHODS

### 2.1 Ethics approval

The Medical Ethics Committee at the University of Anbar, College of Medicine, University of Anbar, Al‐Anbar Governorate, Ramadi, Iraq, has approved this study according to Scientific Research Ethics Committee Book No. 113, which was validated on 4/11/2019. The written informed consent was obtained from all patients and control participants.

### 2.2 Study patients and controls

We looked at Iraqi infertile males with spermatogenesis deficiency who had requested sterility consultations in private infertility clinics. The participants with non‐obstructive azoospermia or severe oligozoospermia were selected. The participants with primary or secondary infertility were enrolled in this study. The term primary infertility used to describe infertility after one year of unprotected sexual contact in couples trying to conceive who have never before been pregnant, while secondary infertility refers to an inability to conceive after a previous pregnancy. A control group of the normal fertile males with a normal spermatogenesis was included in the study. Healthy donors and randomly selected persons were collected from the community. All exclusion criteria are applied to confirm their suitability for this group. There were no signs of gynecomastia. They were married and have offspring and normal seminal parameters. They were healthy fertile men. The selection of azoospermia, severe oligozoospermia patients, and control participants was performed according to the sperm count values stated by the World Health Organization's usual normative standards.^10^ Based on these standards, absence of sperms, sperm count less than 5 million sperms/ml, and sperm count more than 15 million sperms/ml were defined as azoospermia, severe oligozoospermia, and normal state, respectively. Also, the history of continuous psychological stress and medicinal drug consumption was investigated among the studied groups.

### 2.3 Semen analysis

In brief, each research participant's sperm ejaculate was acquired via masturbation at least three days of abstinence. The semen samples were centrifuged for 10 min at 1000 *g*. Finally, a phase‐contrast microscope was used to analyze 20 µl of each sperm sample participate by a special counting chamber.

### 2.4 Molecular investigation

#### 2.4.1 DNA extraction

The SaMag‐12 Automated Nucleic Acids Extraction System (Sacace Biotechnologies, Italy) was used to obtain genomic DNA from 1‐ml whole blood samples in accordance with the manufacturer's instructions.^11^, ^12^

#### 2.4.2 Screening of AZFa microdeletions by multiplex real‐time polymerase chain reaction (real‐time PCR) assay

In multiplex real‐time PCR, the assay was done in two multiplex reactions (Sacace Biotechnologies, Italy) according to the European Academy of Andrology/European Molecular Genetics Quality Network (EAA/EMQN).^13^ All samples were investigated for AZFa gene cluster deletion using sequence‐tagged sites (STSs) markers sY84 and sY86 (Table 1). The SRY (sex determining region on Y) and ZFY (Zinc‐Finger Y) gene‐specific primers were used as the internal controls, and there are two reaction: reaction A consisted of (sY86 STSs, and ZFY/X as an internal control) and reaction B consisted of (sY84 STS, and SRY as an internal control) Based on the recommendations of the EAA/ EMQN, the criteria of complete AZFa deletion in real‐time PCR method are depend on the deletion of both sequence tag sites STS sY86 and sY84, while partial deletion is the lack of one and presence of another.^13^ The whole reaction volume of each multiplex real‐time PCR was 35 μl including 7 μl of DNA sample, 25 μl of Master Mix, and 0.5 μl of each primer. The cycling settings were 94°C for 90 s, followed by 45 cycles of 94°C for 15 s, 64°C for 40 s, and 72°C for 40 s. Fluorescence information from the 3 corresponding channels was collected at the end of the annealing step in FAM/Green, JOE /Yellow /HEX, and ROX/Orange fluorescence channels.

**TABLE 1:** STS sequence of the multiplex real‐time PCR and PCR primers for AZFa gene microdeletion

Locus	STS sequence (5′to 3′)	Size (bp)
*MIX A*		
SRY	sY14‐F‐GAATATTCCCGCTCTCCGGA sY14‐R‐GCTGGTGCTCCATTCTTGAG	472
ZFY	ZFX/Y‐F‐ACCRCTGTACTGACTGTGATTACAC ZFX/Y‐R‐GCACYTCTTTGGTATCYGAGAAAGT	495
AZFa	sY86‐F5′‐GTG ACACACAGACTATGCTTC sY86‐R5′‐ACACACAGAGGGACAACCCT	318
*MIX B*		
SRY	sY14‐F‐GAATATTCCCGCTCTCCGGA sY14‐R‐GCTGGTGCTCCATTCTTGAG	472
ZFY	ZFX/Y‐F‐ACCRCTGTACTGACTGTGATTACAC ZFX/Y‐R‐GCACYTCTTTGGTATCYG AGAAAGT	495
AZFa	sY84‐F‐AGA AGG GTC CTGAAA GCAGGT sY84‐R‐GCCTACTACCTGGAGGCTTC	326

Primers	Sequences	Annealing	Product size
sY14‐F	5′‐GAATATTCCCGCTCTCCGGA‐3′	57°C	472
sY14‐R	5′‐GCTGGTGCTCCATTCTTGAG‐3′		472
sY82‐F	5′‐ATCCTGCCCTTCTGAATCTC‐3′	56°C	264
sY82‐R	5‐CAGTGTCCACTGATGGATGA‐3′		264
sY1064‐F	5′‐GGGTCGGTGCACCTAAATAA‐3′	56°C	110
sY1064‐R	5′TGCACTAAAGAGTGATAATAAATTCTG‐3′		110
sY1182‐F	5′‐ATGGCTTCATCCCAACTGAG‐3′	56°C	247
sY1182‐R	5′‐CATTGGCCTCTCCTGAGACT‐3′		247
sY88‐F	5‐TTGTAATCCAAATACATGGGC‐3′	56°C	123
sY88‐R	5‐CACCCAGCCATTTGTTTTAC‐3′		123

#### 2.4.3 Screening of AZFa sub‐region microdeletions by conventional PCR

All the patients who were diagnosed with AZFa microdeletion by classical real‐time PCR technique were tested with additional primers (sY82, sY88, sY1064, and sY1182) by conventional PCR to decide whether the microdeletion was partial or complete. Based on the EAA/EMQN recommendations, the criteria of complete AZFa deletion in PCR method were depend on the presence of sY82 for the start sub‐region of AZFa and sY88 for the end AZFa sub‐region, while deletion of the both sY1065 for the part near to the start sub‐region (proximal part) and sY1182 for the part near to the end region (distal part). The partial deletion was the presence of both the sY82 and sY88 and deletion of one of sY1065 or sY1182.^13^ The primers were requested and supplied by Alpha DNA Company, Canada, in a lyophilized form, which was dissolved with sterile distilled water to give the final concentration of each primer in 100 pmᴏl/μl (Table 1). The PCR reaction kit (premix) from the Korean biotech company, BIONEER, was selected. The PCR reaction was carried out in a 20 μl solution holding 10 μl premix: Taq DNA polymerase, 250 μM (each) dATP, dGTP, dCTP, dTTP, and 1.5 Mm MgCl2, reaction buffer (PH 9), and loading dye buffer (blue dye), 2 μl of each amplification primers, 5 μl of target DNA, and 11‐μl nuclease‐free water.

The DNA was amplified by conventional PCR/Thermal cycler (ESCO, Canada), and the reaction situations for the markers were set at initial denaturation at 95°C for 15 min for sY14 internal control, 95°C for 3 min for sY82, sY88, sY1064, and sY1182, and then 35 cycles with denaturation at 94°C for 30 s for all markers, annealing at 57°C for 90 s for sY14, 56°C for 30 s for sY82, sY88, sY1064, and SY1182, then extension at 72°C for 60 s for sY14, 72°C for 45 s for sY82, sY88, sY1064 and sY1182, with final elongation at 72°C for 10 min for sY14, and 72°C for 7 min for sY82, sY88, sY1064, and sY1182.

#### 2.4.4 Preparation of agarose gel with ethidium bromide

The PCR amplicons were identified using agarose gel electrophoresis and then viewed with an ultraviolet transilluminator to reveal the specific bands. Tris‐borate acid‐EDTA (TBE) buffer (10×) was diluted 10 times (1×) by mixing 100 ml of TBE buffer (10×) with 900 ml of distilled water. Then, 2% agarose gel was prepared by dissolving 2 g of agarose powder in 100 ml of 1× TBE buffer until the solution became clear. The solution was cooled to under 50°C, and 1 μl of ethidium bromide staining solution was added to make 100 ml of red DNA dye, which was mixed well and poured into the gel chamber. The comb was placed in the chamber and left to cool for 30 min. The DNA ladder 100 bp kit designed by Korean company BIONEER was used to identify the size of double‐stranded DNA from 100–2000 base pairs. Five µl of the 100 bp DNA ladder was loaded into the first well. Ten μl of the amplified DNA samples was loaded into the wells of the gel pocket, and the gel tray was put into the chamber together with 1× TBE buffer. The electrophoresis was then carried out with the following conditions: 5 volt/cm, 100 watts, for 75 min. After the electrophoresis was completed, the gel was put on a UV transilluminator. Finally, a digital picture was made for the evaluation and documentation of the results.

### 2.5 Statistical analysis

The SPSS statistical software (statistical package for the social sciences) version 22.0 (IBM Corporation, Armonk, NY, USA) was used to analyze all data. *p*‐values of 0.05 were used to determine statistical significance.^14^, ^15^

## 3 RESULTS

### 3.1 Patients and controls

In total, 75 Iraqi infertile men with spermatogenesis deficiency (40 men with non‐obstructive azoospermia and 35 men with severe oligozoospermia) and a control group of 25 fertile males with a normal spermatogenesis were selected based on the semen analysis results. The mean ± SD of age for patients and control was 32.91 ± 8.0 and 32.16 ± 5.3, respectively. Overall, 55 (73.3%) and 20 (26.7%) participants had primary and secondary infertility, respectively (Table 2). The severe oligozoospermia cases were significantly higher than azoospermia cases in secondary infertile group, while in the primary infertile group, the opposite was observed. There was a significant correlation between the infertility types and the study groups, based on the Crosstab chi‐Squared tests (*χ* ^2^ = 111.729), *p*‐value < 0.001. Also, there was a significant difference between the infertile and control groups in term of the presence of continuously psychological stress and history of medicinal drug consumption (Table 2). These two items were existed in more than 50% of infertile males, while no one of participants in the control group had them.

**TABLE 2:** Classification of types of infertility in studied groups and their continuously psychological stress and drug consumption history

Groups		Type of infertility		Total
		Primary	Secondary	
Azoospermia	Count	35	5	40
	% within group	87.5%	12.5%	100.0%
Severe oligozoospermia	Count	20	15	35
	% within group	57.1%	42.9%	100.0%
Total	Count	55	20	75
	% within group	73.3%	26.7%	100.0%

Groups		Continuously psychological stress		*p*‐Value	Drug consumption history		*p*‐Value
		No	Yes		No	Yes	
Azoospermia	Count	21	19	<0.00001	16	24	<0.00001
	% within group	52.5%	47.5%		40.0%	60.0%	
Severe oligozoospermia	Count	14	21		19	16	
	% within group	40.0%	60.0%		54.3%	45.7%	
Control	Count	25	0		25	0	
	% within group	100.0%	0.0%		100.0%	0.0%	

### 3.2 AZFa microdeletions using real‐time PCR

The results of AZFa microdeletions using real‐time PCR are summarized in Table 3. Among all infertile patients and the control group, the amplification of SRY and ZFY genes was normal. Out of 75 infertile males, 32 (69.6%) had partial deletion, while 14 (30.4%) males had complete deletion. All normospermic men were screened for AZFa microdeletions, and no one had these microdeletions. The frequency of microdeletions was significantly different between the infertile and control group (*p*‐value = 0.00001).

**TABLE 3:** Proportion of AZFa microdeletions in the studied groups using real‐time PCR

Type of infertility	The proportion *N* (%)	Complete deletion *N* (%)	Partial deletion *N* (%)	sY84 deletion *N* (%)	sY86 deletion *N* (%)	sY84/sY86 deletion (%)	Total *N* (%)
Primary infertile azoospermia	26 (89.7)	8 (30.8)	18 (69.2)	7 (26.9)	11 (42.3)	8 (30.8)	29 (63.0)
Secondary infertile azoospermia	3 (10.3)	0 (0.0)	3 (100.0)	2 (66.7)	1 (33.3)	0 (0.0)	
Primary infertile severe oligozoospermia	10 (58.8)	4 (40.0)	6 (60.0)	5 (50.0)	1 (10.0)	4 (40.0)	17 (37.0)
Secondary infertile severe oligozoospermia	7 (41.2)	2 (28.6)	5 (71.4)	3 (42.9)	2 (28.6)	2 (28.6)	
Total	46 (61.3)	14 (30.4)	32 (69.6)	17 (37.0)	15 (32.6)	14 (30.4)	46 (61.3)

### 3.3 Semen analysis

The proportion of AZFa microdeletions appeared higher in azoospermia men (72.5%, *n* = 29/40) than severe oligozoospermia men (48.6%, *n* = 17/35). Also, the incidence of microdeletions was higher in the primary infertile azoospermia and primary infertile severe oligozoospermia than secondary infertile azoospermia and secondary infertile severe oligozoospermia, respectively (Table 3).

### 3.4 AZFa sub‐region microdeletions using conventional PCR

**FIGURE 1:**
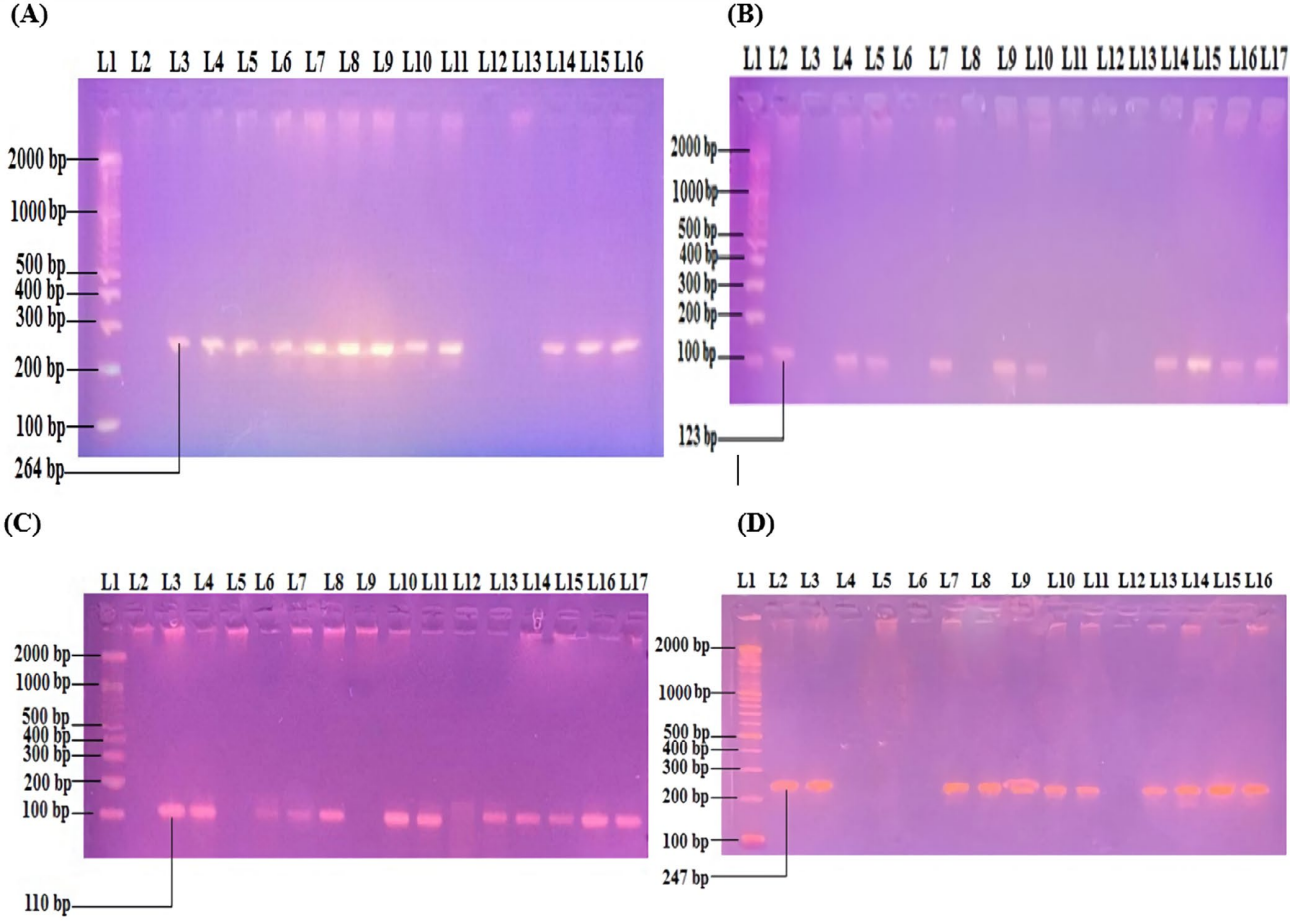
(A) Microdeletion in the sY82 sub‐region of AZFa in azoospermia and severe cases of oligozoospermia. No deletion: L3, L4, L5, L6, L7, L8, L9, L10, L11, L14, L15, and L16; Deletion: L2, L12, and L13; DNA ladder 100 bp: L1. (B) The microdeletion in the sY88 sub‐region of AZFa in azoospermia and severe cases of oligozoospermia. No deletion: L2, L4, L5, L7, L9, L10, L14, L15, L16, and L17; Deletion: L3, L6, L8, L11, L12, and L13; DNA ladder 100 bp: L1. (C) The microdeletion in the sY1064 sub‐region of AZFa in azoospermia and severe cases of oligozoospermia. No deletion: L3, L4, L6, L7, L8, L10, L11, L13, L14, L15, L16 and L17; Deletion: L2, L5, L9 and L12; DNA ladder 100 bp: L1. (D) The microdeletion in the sY1182 sub‐region of AZFa in azoospermia and severe cases of oligozoospermia. No deletion: L2, L3, L7, L8, L9, L10, L11, L13, L14, L15, and L16; Deletion: L4, L5, L6, and L12; DNA ladder 100 bp: L1

This part of the study involved 46 patients including both azoospermic and severely oligospermic men who had previously been screened by classical real‐time PCR and had revealed AZFa microdeletions. For this part, we used additional primers including sY82 for the start sub‐region of AZFa, sY88 for the end AZFa sub‐region, sY1065 for the part near to the start sub‐region (proximal part), sY1182 for the part near to the end region (distal part), and sY14 for internal control. Out of these 46 patients, only one primary infertile azoospermia patient (2.2%) was shown to have complete AZFa deletion, while the other 45 patients (97.8%) had partial AZFa deletions. Amplification of sY82, sY88, sY1064, and sY1182 by a PCR and electrophoresis using 2% a*g*arose gel revealed the bands of amplified targets within the AZFa sub‐region, as shown in Figure 1(A–D). Also, there was no significant association among the previous history of continuously psychological stress and medicinal drug consumption with AZFa microdeletion in infertile males (Table 4).

**TABLE 4:** Association of continuously psychological stress and medicinal drug consumption with AZFa microdeletion in infertile males

Previous history of underlying factors	Infertile male with AZFa microdeletion	Infertile male without AZFa microdeletion	*p*‐Value
Continuously psychological stress	20	20	1.000
Medicinal drug consumption	22	18	0.527

## 4 DISCUSSION

Y chromosome microdeletion in the AZF region is a common genetic source of infertility in males that may take place in three different sub‐regions AZFa, AZFb, and AZFc.^3^ Also, the comicrodeletions in aforesaid sub‐regions may be occurred. Y chromosome microdeletions are often diagnosed worldwide in a variable percentage of infertile men.^16^ This study focused on the frequency of AZFa microdeletions in the Y chromosome and defined the types of AZFa microdeletions among infertile Iraqi men.

Out of 75 infertile men, 55 (73.3%) were patients with primary infertility, while the other 20 (26.7%) were patients with secondary infertility. Our results were nearly in agreement with Öztekin et al.,^17^ from Turkey, who recorded 77.3% and 22.7% for primary and secondary infertility, respectively. In another study from Saudi Arabia, 78.9% of participants had primary infertility.^18^ However, a recent systematic review and meta‐analysis from Africa indicated an approximately same rates for primary and secondary infertilities.^19^ Infertility may be a result of a reproductive tract infection due to sexually transmitted diseases (STDs).^18^ The purpose of this study was not to look for STDs among our patients, but rather to detect the partial and/or complete Y chromosome microdeletions of AZFa sub‐region in infertile Iraqi patients with azoospermia and severe oligozoospermia. After Klinefelter syndrome, Y chromosome microdeletions are the second most common cause of male infertility with a frequency of 2%–50% that lead to defects in spermatogenesis.^20^

In this study, out of 75 infertile patients, 40 (53.3%) infertile males suffered from constant psychological stress, as did 19 azoospermic males (47.5%) and 21 males with severe oligozoospermia (60.0%). The infertile men were concerned about society's view of their infertility and also about environmental factors and aging. Some of them were receiving treatment secretly for fear of how society would view them and this meant that they were preoccupied and in a state of constant anxiety, in addition to the economic and security conditions that society was experiencing. Indeed, several studies have reported an association between the quality of semen and the presence of lifestyle stressors and the negative impact of psychological stress on the quality, morphology, and quantity of semen fluid leading to infertility.^21^, ^22^ A study also showed an increase in cases of infertility in Iraq in the period between 1980 and 2013. Whereas from 1980–1990, the number of infertile males identified was 224, and from 2003 to 2013, this rose to 2740, an increase which may be due to environmental pollution, psychological stress, and wars.^23^

The present study has been able to include more information about the occurrence of AZFa in Iraq, which was rarely documented previously. In our region, we found a much higher frequency of AZFa microdeletions than several previous studies from different countries. Out of 75 infertile males with azᴏᴏspermia and severe oligozoospermia, the frequency of AZFa microdeletions was shown to be 61.3% (46 patients). In the previous studies by Al‐Janabi et al.^9^ and Hanoon et al.^24^ from Iraq, the AZFa microdeletions were not detected in any patients that was in contrast with the current research. These studies reported the AZFc as the most prevalent microdeletion in Iraq followed by AZFb.^9^, ^24^ Also, in a previous study from Iran, no AZFa microdeletion was identified.^25^ However, in a study from Sudan, the most frequent microdeletion was found in the AZFa region (11 out of 30 patients, 71.4%), followed by the AZFc (*n* = 4) and the AZFb (*n* = 3).^26^ Dutta et al.^27^ from India found the AZFa microdeletions in 4.2% of infertile patients that was lower than our results.

The diversity of these results may be due to different numbers of STS primers being used to diagnose microdeletions, differences in the targeted criteria used for recruitment of infertile patients, different methodologies used to determine the frequencies, the size of the study samples, environmental influences, and the fact that the studies were carried out on diverse ethnic populations.^12^, ^25^, ^27^

Infertile men with severe azoospermia/oligozoospermia usually use sperm retrieval techniques such as microsurgical testicular sperm extraction (microTESE) for treatment. To predict the probability of microTESE success, AZF microdeletions are often screened before surgery. There is virtually no chance of this happening in cases with AZFa and AZFb microdeletions.^28^ Since premarital genetic counselling has not yet been formally approved in Iraq, little is known about the prevalence of chromosomal microdeletions leading to infertility. Therefore, due to the high prevalence of microdeletions in our region, it is recommended that chromosome microdeletions screening be performed in premarital counselling. Also, it is recommended that these microdeletions be screened in infertile Iraqi males before surgeries to avoid incurring excessive costs to the patient.

In this study, the prevalence of AZFa microdeletions was higher in azoospermia patients than those with severe oligozoospermia. These findings were in agreement with the findings of Dutta et al.^27^ and in contrast with the results of Elsaid et al.^26^ Also, in line with our findings, Sha et al.^28^ from China, Asadi et al.^30^ from Iran, and Kim et al.^31^ from Korea, reported higher prevalence of AZFa microdeletions in patients with azoospermia than in those with oligozoospermia. Although in this study, AZFa microdeletion was found in both groups, but in previous studies from Egypt,^16^, ^32^ India,^27^ China,^29^ Iran,^30^ and Korea,^31^ it was identified only in the azoospermia patients, which was contrary to this research.

This study found that infertile individuals had significantly more microdeletions than control individuals (*p*‐value = 0.00001), suggesting that these microdeletions are associated with infertility. These findings were in line with previous studies from Sudan^26^ and India.^27^ Also, in this study, the AZFa microdeletion in infertile males was not associated with the previous history of psychological stress or drug consumption. However, the infertile and control groups differed significantly in their histories of psychological stress and pharmaceutical consumption. There is growing evidence that male reproduction can be influenced by environmental factors, psychological factors, genetic factors, dietary habits, socioeconomic status, and drug abuse.^33^, ^34^, ^35^ However, there has not yet been a comprehensive investigation of the exact role of AZF regions in male infertility. Therefore, more studies are needed before they can be applied to clinical field.^36^

Finally, the current study confirmed that to determine the complete or partial deletion of AZFa region, more STS are needed be used and the two STS sY86 and sY84 alone are not enough to identify the microdeletion type. This is important because in patients with a complete AZFa deletion, ICSI or microTESE is not recommended as an assisted productive treatments.^5^, ^28^, ^36^ Based on the EAA/EMQN recommendations, all azoospermia or severe oligozoospermia patients have the indications to perform the Y chromosome microdeletion screening.^13^ Today, more advanced techniques such as array comparative genomic hybridization (aCGH) and multiplex ligation‐dependent probe amplification (MLPA) have been introduced to investigate infertility‐related chromosomal abnormalities, because the STS‐PCR method cannot determine all AZF‐linked microdeletions, duplications, or complex copy number variants (CNVs). This method mainly detects the AZF‐linked deletions.^37^

This study had several limitations as follows: due to limited budget and financial resources, AZFc and AZFb microdeletions were not investigated. Another limitation of this study was the small sample size due to traffic restrictions caused by the coronavirus disease 2019 (COVID‐19) pandemic. Because of this pandemic, we could not recruited healthy individuals as control group. As you know, several researches have been affected by the mentioned pandemic in the past two years. Also, the levels of reproductive hormones including follicle stimulating hormone (FSH) and luteinizing hormone (LH) were not assayed. Moreover, the karyotyping and evaluation of testicular biopsies were not performed.

## 5 CONCLUSION

This study showed a high frequency of AZFa microdeletions in Iraq compared with several countries. In our study population, partial AZFa microdeletions were higher than complete AZFa deletion. We needed to study each sub‐region in detail by using many primers, not only the STS primers recommended by EAA/EMQN, to judge whether the type of deletion was complete or partial. The results of this study indicated the necessity of Y chromosome microdeletion screenings for male infertility diagnosis in Iraq. For assisted reproductive techniques, it is crucial to obtain accurate genetic information to minimize the unnecessary treatments and the vertical inheritance of genetic defects to the next generation.

## CONFLICT OF INTEREST

The authors declared that they have no competing interests.

## Data Availability

The datasets used and/or analyzed during the current study are available from the corresponding author on reasonable request.
